# Successful total en bloc spondylectomy of the L3 vertebra with a paravertebral giant cell tumor following preoperative treatment with denosumab: a case report

**DOI:** 10.1186/s13256-019-2029-4

**Published:** 2019-04-26

**Authors:** Hideyuki Kinoshita, Sumihisa Orita, Tsukasa Yonemoto, Takeshi Ishii, Shintaro Iwata, Hiroto Kamoda, Toshinori Tsukanishi, Kazuhide Inage, Koki Abe, Masahiro Inoue, Masaki Norimoto, Tomotaka Umimura, Kazuki Fujimoto, Yasuhiro Shiga, Hirohito Kanamoto, Takeo Furuya, Kazuhisa Takahashi, Seiji Ohtori

**Affiliations:** 1 Department of Orthopaedic Surgery, Graduate School of Medicine, Chiba University, 1-8-1, Inohana, Chuo-ku, Chiba, 260-8670 Japan; 2 Department of Orthopaedic Surgery, Saiseikai Narashino Hospital, 1-1-1, Izumicho, Narashino, Chiba 275-8580 Japan; 3 Department of Orthopedic Surgery, Chiba Cancer Center, 666-2 Nitonacho, Chuo-ku, Chiba, 260-8717 Japan; 4 Department of Musculoskeletal Oncology, National Cancer Center Hospital, 5-1-1, Tsukiji, Chuo-ku, Tokyo, 104-0045 Japan; 5 Department of Orthopedic Surgery, Tsukuba University, 1-1-1, Tennodai, Tsukuba, Ibaraki 305-8577 Japan

**Keywords:** Giant cell tumor, Denosumab, Total en bloc spondylectomy, Paravertebral lesion

## Abstract

**Background:**

Giant cell tumor is known to be a benign neoplasm that arises most commonly in the long bones, while cases in the spine are rare. Recently, denosumab, a monoclonal antibody that inhibits receptor activator of nuclear factor-kappa β ligand, has been used to treat patients with giant cell tumor. However, there are few reports of total en bloc spondylectomy being used for paravertebral giant cell tumor lesions following denosumab therapy.

**Case presentation:**

Our patient was a 20-year-old Japanese woman with a 4-month history of lower back pain. A spinal computed tomography scan and magnetic resonance imaging of her lumbar spine revealed an osteolytic lesion involving the L3 vertebral body, and the tumor extended toward the left side of the paravertebral soft tissue and into the left pedicle. The lesion was diagnosed as a giant cell tumor by needle biopsy. Denosumab treatment calcified the paravertebral giant cell tumor lesion and the tumor vertebral body was removed completely by total en bloc spondylectomy.

**Conclusion:**

This case report describes a patient with a paravertebral giant cell tumor who was successfully treated by preoperative denosumab injection followed by total en bloc spondylectomy.

## Background

Giant cell tumors (GCTs) account for 4–8% of all primary bone tumors, and they are most frequently located in the juxta-articular metaphysis of the long bones [1], while 2–3% of GCTs develop in the spine and sacrum [2]. Denosumab, a monoclonal antibody that inhibits receptor activator of nuclear factor-kappa β ligand (RANKL), is used in patients with GCT, inhibiting osteoclast-like giant cell recruitment, thereby preventing the osteolysis typical of GCT [3]. Treatments for GCT depend on its location; excision and intralesional curettage are used for GCTs in the extremities, whereas denosumab followed by total en bloc spondylectomy (TES) is used for spinal GCTs. TES may be difficult in some patients, however, because of the fragility or paravertebral location of GCT. This case report describes a patient with a paravertebral GCT who was successfully treated by TES of the L3 vertebra following denosumab therapy.

## Case presentation

A 20-year-old Japanese woman with a 4-month history of severe lower back pain was referred to our out-patient department. She had no history of fever, trauma, weight loss, or previous infection.

**Fig. 1:**
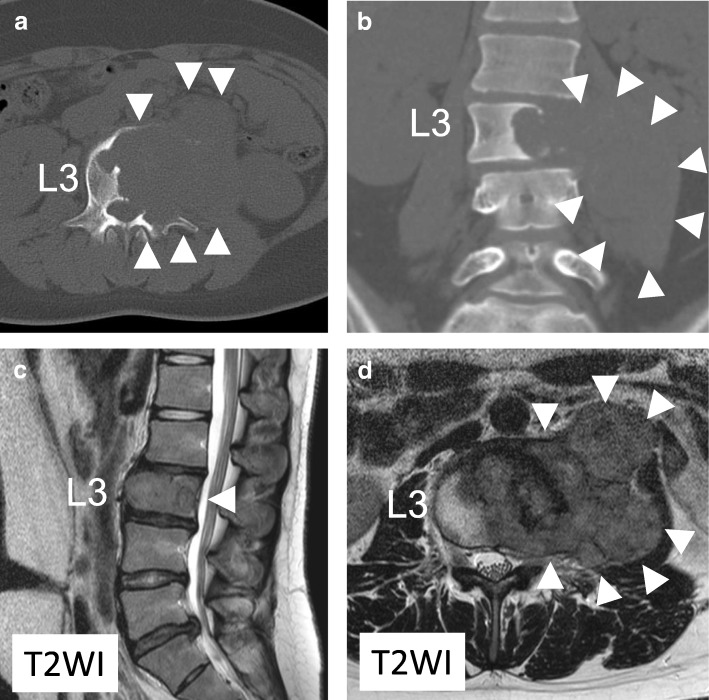
**a**, **b** Computed tomography prior to denosumab treatment, showing an osteolytic lesion involving the L3 vertebral body, which collapsed and expanded into the left side of the iliopsoas muscle (*arrowhead*). **c**, **d** T2-weighted magnetic resonance images prior to denosumab therapy, showing the tumor extending toward the left side of the paravertebral soft tissue (*arrowhead*). *T2WI* T2-weighted image

**Fig. 2:**
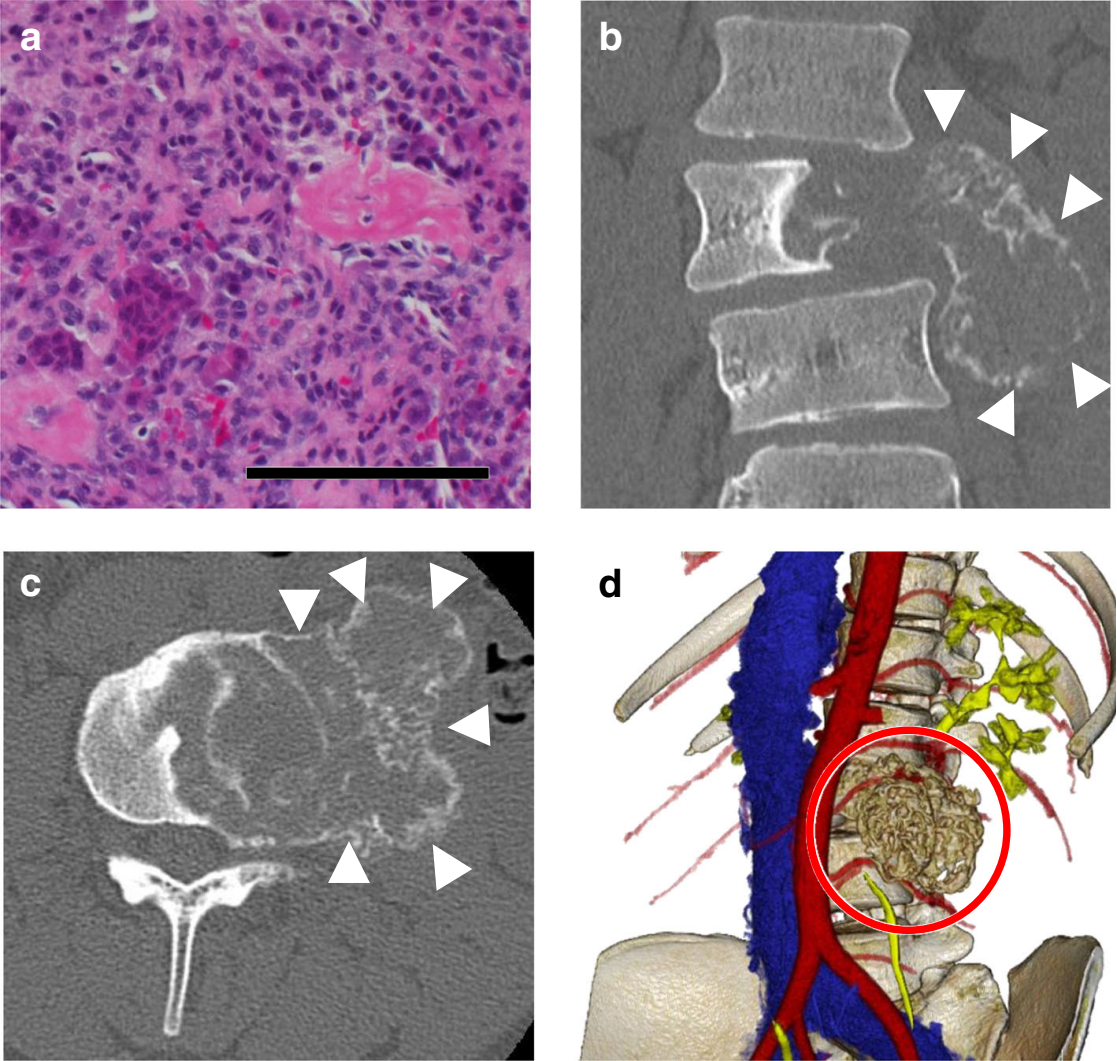
**a** Histologic examination of a tumor biopsy specimen prior to denosumab therapy, showing multinucleate giant cells surrounded by neoplastic stromal cells (hematoxylin and eosin staining; scale bar, 100 μm). **b-c** Computed tomography images after 6 months of denosumab treatment show a clear border between the vertebral tumor body and soft tissue, indicating calcification of the tumor body (*arrowhead*). **b** Coronal view. **c** Axial view. **d** Three-dimensional reconstructed image from enhanced computed tomography

Radiographic analysis showed collapse of the left side of the L3 vertebral body and swelling of the iliopsoas muscle. Spinal computed tomography (CT) revealed an osteolytic lesion involving the L3 vertebral body and surrounding soft tissue, causing vertebral body collapse (Fig. 1a, b). Magnetic resonance imaging (MRI) of her lumbar spine showed the tumor extending toward the left side of the paravertebral soft tissue and into the left pedicle (Enneking SIII) (Fig. 1c, d). Pathological and immunohistochemical analyses of a needle biopsy specimen showed a GCT with multinucleate giant cells surrounded by neoplastic stromal cells (Fig. 2a). A phase 2 trial showed no adverse effects or complications of denosumab, so she was prescribed six cycles of monthly subcutaneous injections of 120-mg denosumab [4]. Lumbar CT during denosumab treatment showed that the tumor included a paravertebral lesion with progressive calcification (Fig. 2b–d).

**Fig. 3:**
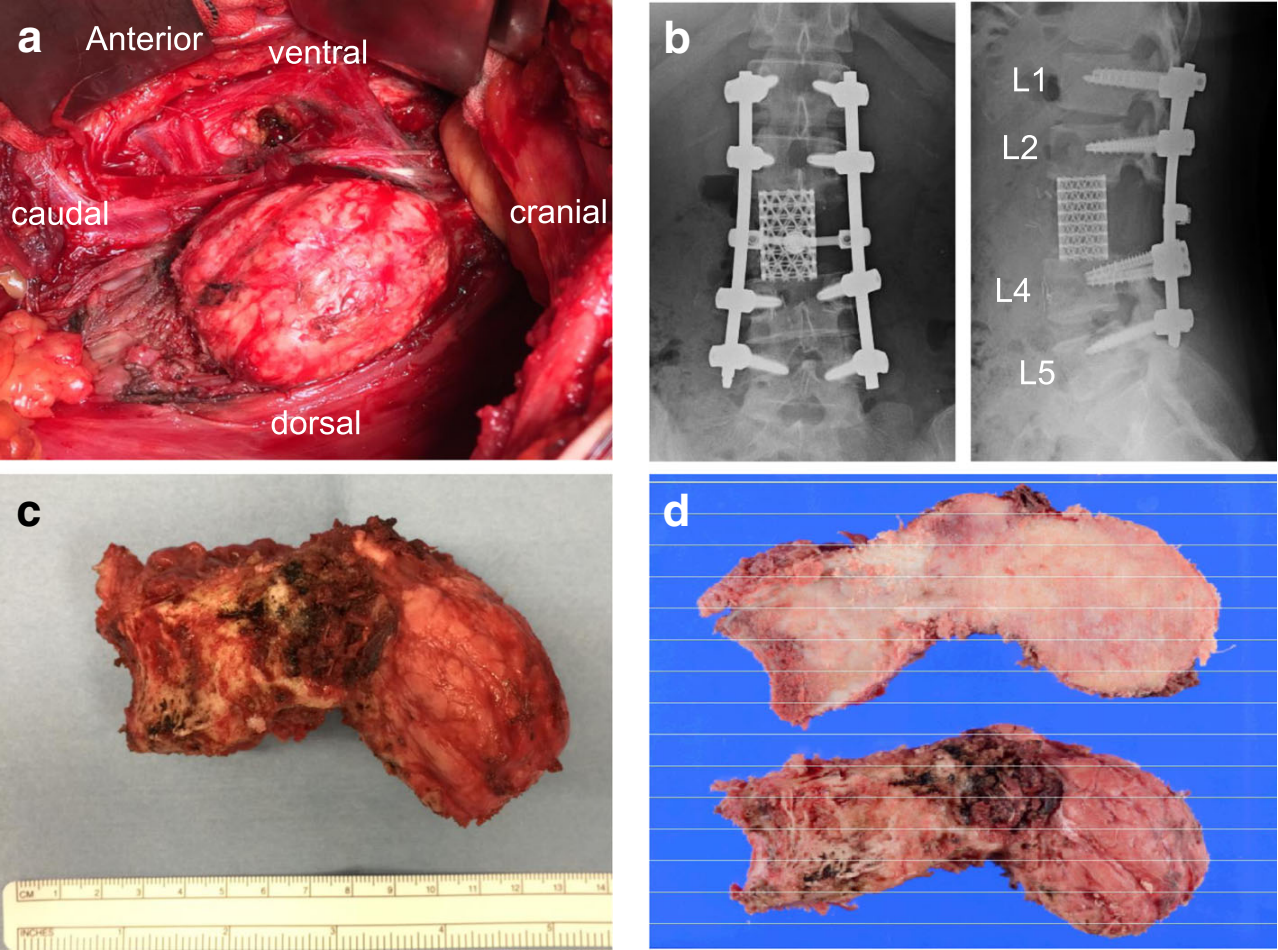
**a** Intraoperative examination of the tumor at anterior retroperitoneal approach. **b** Plain radiographs of the anteroposterior and lateral view. **c**, **d** Results showing that the L3 vertebral tumor body had been completely removed by the anterior transperitoneal approach

Following denosumab treatment, she underwent two-stage (anteroposterior) L3 TES. Stage 1 utilized a posterior approach for resecting the posterior vertebral component; the total operation took 5 hours 16 minutes and the total bleeding was 1520 ml. Stage 2 utilized an anterior retroperitoneal approach for resecting the anterior vertebral component followed by intervertebral cage insertion; the total operation took 6 hours 43 minutes, and the total bleeding was 2320 ml (Fig. 3a, b). The day before the second-stage operation, preoperative angiography and segmental artery embolization from L3 to L4 were performed to reduce intraoperative bleeding. The vertebral body was removed completely after the discectomies, and the bilateral psoas muscle was released from the L3 vertebral body (Fig. 3c, d). There were no complications during or after the surgery. She was discharged on the seventh postoperative day, ambulatory and without neurological deficits. Two years after surgery, she has not experienced GCT recurrence or implant failure.

## Discussion and conclusions

GCT, or osteoclastoma, is a relatively common benign bone tumor, usually arising from the metaphysis of long bones and extending into the epiphysis. Spinal GCT is rare, accounting for < 2–3% of GCTs [5], and its treatment remains challenging. Surgery for GCT traditionally consists of intralesional curettage with local adjuvant treatment (for example, polymethylmethacrylate known as PMMA, liquid nitrogen, phenol) or TES, which is effective for lumbar spine tumors without significant risks of neurological deficits or mortality [6, 7]. Optimal TES candidates are patients with Enneking SIII, an aggressive benign spine tumor [8]. Because local recurrence is common following intralesional curettage with adjuvants, reoperation and/or postoperative radiotherapy may be required [9]. In contrast, TES exhibits rarer local recurrence. Local recurrence rates following intralesional curettage and TES are reportedly 50–80% and 0–15%, respectively [10]. As other studies have reported that TES is effective for thoracic GCT [11, 12], we performed TES for this case of an L3 vertebra with a paravertebral GCT. However, there is no report that TES of huge spinal and paravertebral GCT following preoperative denosumab as in the current case is effective. The current case will be a meaningful report that denosumab is effective even for a huge GCT extending to the soft tissue around the vertebral body.

Denosumab was recently approved for GCT, and for those with severe osteoporosis [13]. Denosumab inhibits the recruitment of osteoclast-like giant cells by neoplastic stromal cells [3], thereby preventing the osteolysis typically seen in GCT. An open-label phase II study (NCT00396279) of denosumab in patients with GCT reported that 86% had objective responses, that is, elimination of > 90% giant cells or absence of radiographic progression of the target lesion [14]. Preoperative treatment of our patient with denosumab successfully induced GCT calcification and facilitated TES of the tumor vertebrae. Although denosumab is effective for osteoporosis and GCT, it has side effects of hypocalcemia. Salim *et al.* reported hypocalcemia in an estimated 8–14% of the patients treated with denosumab and even more frequently among those with chronic kidney disease (CKD) [15]. To prevent the side effect it is important to assess carefully the vitamin D status and parathyroid hormone (PTH) status in patients with CKD before denosumab exposure and to closely monitor serum calcium levels subsequently.

The need for postoperative denosumab is still to be determined. Inhibition of RANKL may increase the risk of new malignancies by inducing immunosuppression [16]. Broehm *et al*. reported two cases of sarcoma that arose from bone GCTs by long-term denosumab administration [17]. On the other hand, there have been cases of GCT recurrence following the cessation of denosumab therapy. Although the previous report showed that the mean time to recurrence was approximately 2 years after surgery [18], there has been no recurrence in the current case 2 years after the operation, suggesting the possibility that TES combined with preoperative denosumab is effective for giant spinal GCT. Furthermore, as the use of denosumab to treat GCT will probably increase, further controlled studies to determine the optimal period to use denosumab without promoting malignant transformation and recurrence are needed.

In conclusion, this case report describes a patient with paravertebral GCT who was successfully treated by preoperative denosumab injection followed by TES.

## Abbreviations

CKD: Chronic kidney disease
CT: Computed tomography
GCT: Giant cell tumor
MRI: Magnetic resonance imaging
PMMA: Polymethylmethacrylate
PTH: Parathyroid hormone
RANKL: Receptor activator of nuclear factor-kappa β ligand
TES: Total en bloc spondylectomy
